# Acute motor-sensory axonal polyneuropathy variant of Guillain-Barré syndrome with a thalamic lesion and COVID-19: a case report and discussion on mechanism

**DOI:** 10.3389/fneur.2023.1227505

**Published:** 2023-09-14

**Authors:** Na Geng, Pengfei Wang, Yong Zhang

**Affiliations:** Department of Neurology, Weihai Municipal Hospital, Weihai, China

**Keywords:** Guillain-Barré syndrome, COVID-19, neuro-electrophysiological characteristics, MRI findings, thalamic lesion

## Abstract

**Background:**

Severe acute respiratory syndrome coronavirus 2 (SARS-CoV-2) primarily affects the respiratory system. During the global coronavirus disease (COVID-19) pandemic, COVID-19-associated neurological diseases have been increasingly reported, including peripheral nervous system diseases, such as Guillain–Barré syndrome (GBS). Acute motor-sensory axonal polyneuropathy (AMSAN), is a GBS variant associated with COVID-19. To date, there are no reports of GBS cases with thalamic injury and dynamic evolution with fluctuating GBS symptoms. In this report, we describe the first case of COVID-19-associated AMSAN accompanied by a thalamic lesion and discuss the magnetic resonance imaging (MRI) findings.

**Case presentation:**

A 76-year-old woman, with known co-morbid type 2 diabetes mellitus, presented to the emergency room with complaints of weakness and paraesthesia in both her legs and arms for 4 days, and fever and dry cough for the past 5 days. A nasopharyngeal swab for SARS-CoV-2 returned positive. The patient had not received specific treatment for COVID-19 infection. Neurological examination disclosed symmetric weakness (Medical Research Council grade upper limbs 4/5, lowers limbs 2/5) and areflexia in both the legs and feet. No cranial nerves were involved. Following a neuro-electro-physiology study to evaluate neurological symptoms, AMSAN was suggested. Cerebrospinal fluid (CSF) analysis showed elevated protein levels that confirmed the diagnosis of GBS. The patient was subsequently treated with intravenous immune globulin (IVIG), which improved her neurological symptoms (upper limbs 4/5, lowers limbs 4/5). However, urinary retention, dysarthria, dysphagia, bilateral facial paralysis, facial diplegia, bucking, and motor alalia gradually appeared, followed by aggravated paralysis (upper limbs 3/5, lowers limbs 1/5). After being hospitalized for 16 days, the patient underwent continuous plasma exchange (PE) treatment for a duration of 3 days. Following treatment, the patient’s neurological symptoms and paralysis gradually improved (upper limbs 4/5, lowers limbs 4/5) over 2 weeks. Meanwhile, we observed that the patient’s cerebral magnetic resonance imaging (MRI) findings dynamically evolved along with the fluctuation of her GBS symptoms, mainly in terms of the changes in T2 hyperintensity in the right thalamus accompanied by microhaemorrhages. The inflammation index was normal. We considered a wide range of possible causes including hypoxia, drugs, toxins, and metabolic derangements but these were excluded.

**Conclusion:**

The AMSAN variant of GBS secondary to COVID-19 infection is severe and can cause extensive damage to the peripheral nerves system. The deterioration of symptoms in the patient after early immunotherapy may indicate treatment-related fluctuation (TRF) and could be attributed to immune rebound. Moreover, an excessive immune response post-COVID-19 infection may trigger concurrent damage to the central nervous system, indicating secondary harm to brain small blood vessels and nerve units. For suspected cases of GBS complicated by COVID-19, it is essential to conduct early brain MRI examinations in addition to routine peripheral nervous system evaluations to promptly detect any intracranial lesions. This facilitates appropriate immunotherapy and improves patient prognosis.

## Introduction

**Figure 1:**
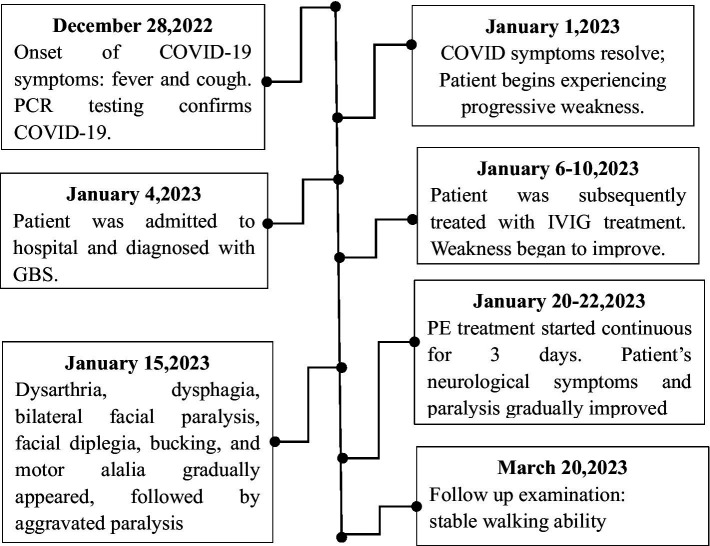
Patient timelines. Starting December 28, 2022, the timeline of the patient’s diagnosis, treatment, recovery and other significant events are outlined until March 20, 2023, the symptoms of weakness recover.

Severe acute respiratory syndrome coronavirus 2 (SARS-CoV-2), which causes the coronavirus disease 2019 (COVID-19), primarily affects the respiratory system. During the global COVID-19 pandemic, neurological complications of COVID-19 have been increasingly reported, including Guillain–Barré syndrome (GBS) (1). GBS is an acute poly radiculo-neuropathy characterized by rapid progression, symmetrical limb weaknesses, areflexia, sensory symptoms, and, in some patients, facial weakness. GBS is a life-threatening inflammatory/autoimmune condition, in which the immune system targets healthy nerve cells in the peripheral nervous system (PNS) (2). The common variants of GBS include acute inflammatory demyelinating polyradiculoneuropathy (AIDP) which is a motor sensory demyelinating disorder; and acute motor axonal neuropathy (AMAN), and acute motor and sensory axonal neuropathy (AMSAN), both of which are axonal disorders (3). The AMSAN variant of GBS has been associated with COVID-19 (4). The symptoms of AMSAN are severe and the prognosis is poor. To date, there are no reported cases of AMSAN involving the central neurovascular unit or discussions relating to the findings on cerebral magnetic resonance imaging (MRI). In this report, we described the first case of COVID-19-associated AMSAN, accompanied by a thalamic lesion detected on MRI. We also discussed the thalamic injury, in which the symptoms dynamic evolved and fluctuated (Figure 1).

## Case presentation

On January 6, 2023, during the COVID-19 epidemic in this region of China, a 76-year-old woman, with known co-morbid type 2 diabetes mellitus, presented to the emergency room of our hospital, the Third Grade First Class Hospital, with complaints of weakness and paraesthesia in both her legs and arms for four days, and fever and dry cough for the past 5 days. A nasopharyngeal swab for SARS-CoV-2 returned positive. The patient had received the inactivated COVID-19 virus vaccine one year ago, and had not been administered any other specific antiviral therapy for a COVID-19 infection.

**Figure 2:**
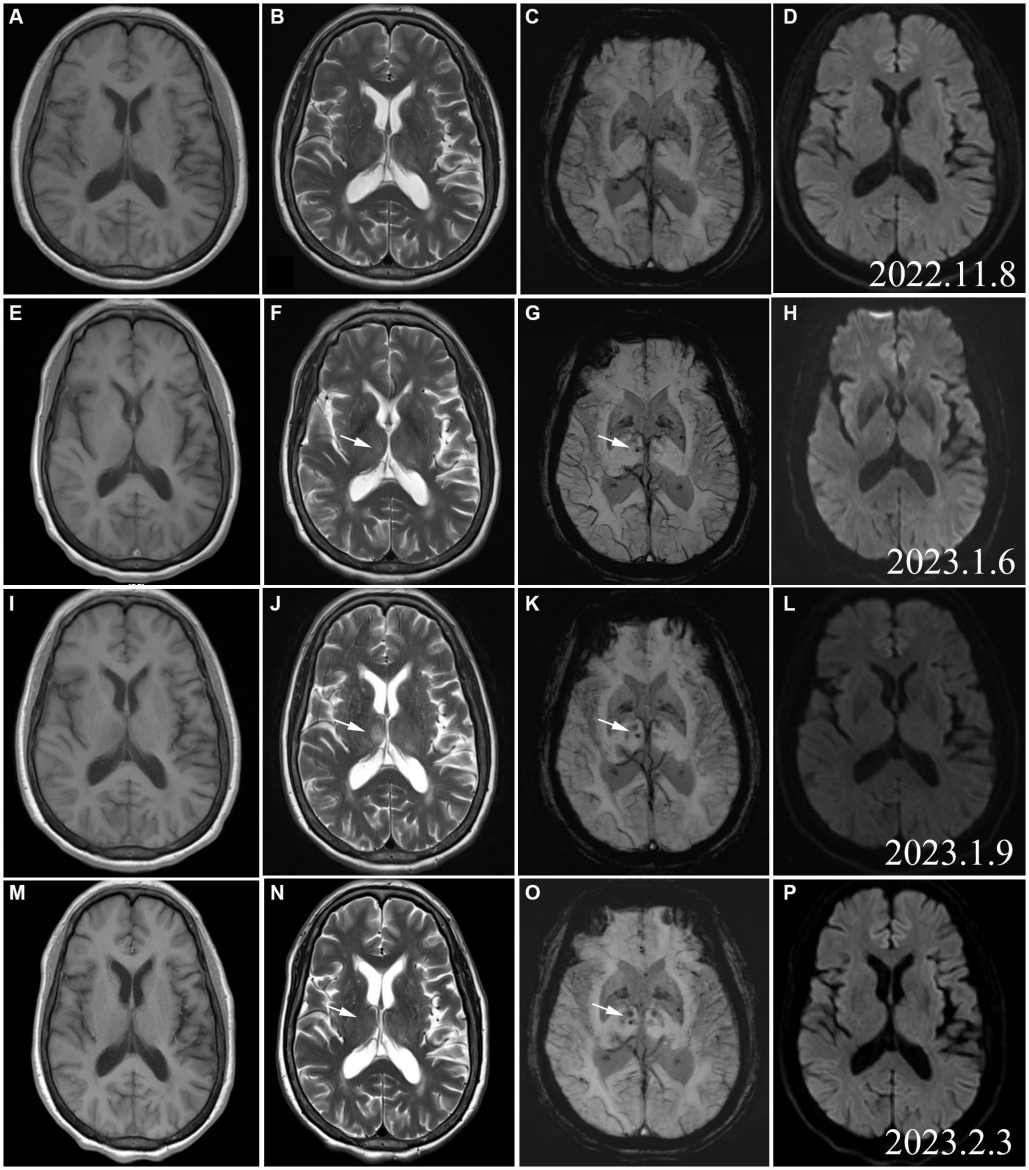
Cerebral MRI (2022.11): **(A)** T1WI; **(B)** T2WI; **(C)** SWI; **(D)** DWI. No signals in the right thalamus on the images acquired with these imaging sequences. MRI **(A–D)** conducted before the onset of the patient, and no abnormal signals were found. As a group of normal controls, we found subsequent changes in thalamic lesions. Cerebral MRI (2023.1.6): E: T1WI; **(F)** T2WI; **(G)** SWI hypointense punctate foci. **(H)** No DWI hyperintensity. Cerebral MRI (2023.1.19): **(I)** T1WI hypointensity in the right thalamus. **(J)** T2WI hyperintensity in the right thalamus; **(K)** SWI hypointense punctate foci in the right thalamus; **(L)** No DWI hyperintensity,represented the signal contrast of the rest of the MR Sequence; **(N)** T2WI hyperintensity in the right thalamus disappeared; **(O)** SWI hypointense punctate foci in the right thalamus; **(P)** No DWI hyperintensity.

On admission, baseline laboratory investigations were performed (Table 1). A nasopharyngeal swab was repeated for COVID-19 PCR testing. The result of which was negative. However, the serologic test result was positive. A brain MR was performed prior to the patient’s onset (2022.11), and no abnormal signals were detected (Figures 2A–D). Following admission, the patient underwent a subsequent cerebral MRI re-examination, which revealed slightly elevated signals (T2) and low signals in the right thalamus (SWI) (Figures 2F,G). However, no hyperintensity was observed in T1 or DWI (Figures 2E,H). The patient was treated with intravenous immunoglobulin (IVIG 0.4 g/kg·d) for 5 days.

**Table 1:** Baseline investigations of the patient on admission.

Parameter	Result	Normal range
*Complete blood count*		
Hb (g/dl)	143	115–150
MCV (fl)	88	82–100
MCH (pg)	30.8	27–34
MCHC (Gm/dL)	350	316–354
Total leukocyte count (*10^9^/L)	1.75	1.1–3.2
Lymphocytes (%)	29.9	20–50
Neutrophils (%)	63.8	40–75
Platelets count (counts/uL)	262,000	125,000–350,000
*Inflammatory markers*		
CRP (mg/dL)	1.98	0–3
ESR (mm/h)	31	0–20
*Biochemical profile*		
Sodium (mEq/L)	135	135–145
Potassium (mEq/L)	3.91	3.5–5.5
Chloride (mEq/L)	97.2	96–108
Calcium (mEq/L)	2.27	2.12–2.52
Magnesium (mg/dl)	0.66	0.66–1.07
Phosphorous (mg/dl)	0.79	0.81–1.58
BUN (mg/dL)	3.94	3.2–7.1
Cr (mg/dl)	54.6	36–100
*COVID-19 antigen*		
IgG (500copy/ml)	149.02	0–1
IgM (500copy/ml)	3.45	0–1
**CSF protein (mg/dL)**	1871.9	150–450
CSF cell (*10^**6**^**/L)**	2	0–1

Hb, Hemoglobin; MCV, Mean Corpuscular Volume; MCH, Mean Corpuscular Hemoglobin; MCHC, Mean Corpuscular Hemoglobin Concentration; CRP, C-Reactive Protein; ESR, Erythrocyte Sedimentation Rate; BUN, Blood Urea Nitrogen; Cr, creatinine; CSF, Cerebrospinal fluid.

On further investigation, the neuro-electrophysiology (Table 2) examination on January 8, 2023 showed a decreased compound muscle action potential (CMAP) amplitude and sensory nerve action potential (SNAP) amplitude in the left median and ulnar nerves. Based on these results, AMSAN was suggested. Cerebrospinal fluid (CSF) analysis was performed and the results demonstrated elevated protein levels (1871.9 mg/dL, normal 150–450 mg/dL) while the leukocyte level was normal (2/mm3), Based on the clinical findings, neurophysiological studies, CSF analysis, and cervical MRI, the patient was diagnosed with AMSAN, a GBS variant associated with COVID-19. The MRI of the patient’s cervical spine revealed no abnormal signals in the cervical spinal cord, ruling out the possibility of limb numbness and weakness caused by lesions in the cervical spinal cord. Although we considered COVID-19-related AMSAN based on patients’ history of COVID-19 infection, acute limb movement, sensory disturbance, protein cell isolation, and EMG results, there are limitations due to the lack of proximal CMAPs and the timing of the EMG study. Furthermore, despite the patient’s history of diabetes, there were no neuropathic symptoms (sensory or motor) related to diabetes. Therefore, considering the distal axonal sensory and motor changes caused by AMSAN.

**Table 2:** Neuro-electrophysiology report results.

*Motor nerve conduction study*								
Nerve recording site	CMAPS amplitude (mV)		Distal latency (ms)		Proximal latency (m/s)		Conduction velocity (m/s)	
	Lt.	Normal value	Lt.	Normal value	Lt.	Normal value	Lt.	Normal value
Median APB	2.7	>4	8.78	<4.5	14.4		39.1	45–55
Ulnar ADM	5.0	>6*	4.31	<4.2	3.27		58.6	45–55
Peroneal EDB	0.044	>2	9.46	<6.5	17.4		33.3	40
Tibial AHB	0.74	>3	5.19	<6.5	16		30.7	40
Peroneal TA	0.080	>2	7.6	<3	28.4		36.5	40

Sensory nerve conduction study				
Nerve	Latency		Snaps Amplitude UV	
	Recording value	Normal range	Recording value	Normal range
Left Median	3.72	2.9–3.5	0.45	>15
Left Ulnar	2.97	2.9–3.5	7.7	>15
Left Sural	4.15	<4.2	3.8	>5

APB, Abductor pollicis brevis; ADM, abductor digiti minimi; EIP, extensor indicis proprius; EDB, extensor digitorum brevis; AHB, abductor halluces brevis; TA, tibialis anterior; *<70 years old, the baseline reference value for CAMP is > 4, >70 years old, the baseline diagnostic data for CMAP is > 6 (Stetson DS, JW. Albers. Effect of age, sex, and anthropometric factors on nerve conduction measures. Muscle Nerve, 1992,15:1096–1,104).

After some improvement of the limb weakness following treatment with IVIG, the patient experienced aggravated symptoms including choking on water, slurred speech, weakness during chewing, reduced muscle strength, and urinary retention. The neurological examination on day 11 reported the patient as conscious, with dysarthria, bilateral eyelid closure weakness, a left eye fissure of 6 mm and a right eye fissure of 4 mm, a flattened left nasolabial fissure, tongue deviation towards the right side on attempted protrusion, grade 3 upper limb muscle strength, grade 1 lower limb muscle strength, negative patellar reflex (−), and negative Babinski sign (−). The above symptoms continued to aggravate. On January 19, 2022, a re-examination with cerebral MRI revealed that the right thalamus had T1 hypointensity and a more pronounced T2 hyperintensity than before. Moreover, hypointensity was detected on the susceptibility-weighted imaging (SWI), while no hyperintensity was noted on the diffusion-weighted imaging (DWI) (Figures 2I–L). After 16 days of hospitalization, the patient underwent a continuous plasma exchange (PE) treatment for a duration of 3 days. On February 3, 2022, the patient had grade 4 muscle strength in the upper limbs and grade 2 muscle strength in the lower limbs. Another re-examination with cerebral MRI revealed a loss of both T1 hypointensity and T2 hyperintensity in the right thalamus. Only the hypointense lesion on SWI was visible (Figures 2M–P). Two weeks after the follow-up, the lower limb muscle strength of the patient improved to grade 4. The patient continued rehabilitation by OT/PT cotreatment sessions (Table 3). One month after the follow-up, the patient demonstrated stable walking ability and overall improvement of her condition.

**Table 3:** Upper and lower extremity assessments (MRC scores).

Assessment	Admission	Progression	Discharge
*RUE strength*			
Shoulder	4−/5	3/5	4−/5
Elbow	4−/5	3/5	4−/5
Wrist	4/5	3+/5	4/5
Grip	4/5	3+/5	4/5
*LUE strength*			
Shoulder	4−/5	3/5	4−/5
Elbow	4−/5	3/5	4−/5
Wrist	4/5	3+/5	4/5
Grip	4/5	3+/5	4/5
*RLE strength*			
Hip flexion	2/5	1+/5	4−/5
Hip	2/5	1+/5	4−/5
Knee	2/5	1+/5	4−/5
Ankle dorsiflexion	2/5	1+/5	4−/5
*RLE strength*			
Hip flexion	2/5	1+/5	4−/5
Hip	2/5	1+/5	4−/5
Knee	2/5	1+/5	4−/5
Ankle dorsiflexion	2/5	1+/5	4−/5

RUE, right upper extremity; LUE, left upper extremity; RLE, right lower extremity; LLE, left lower extremity.

## Discussion

Several variants of GBS exist. It is an immune-mediated polyneuropathy that is most often preceded by an infection. In this report, we described a patient with AMSAN, a variant of GBS, that presented with a thalamic lesion, and was associated with COVID-19.

AMSAN is more commonly associated with COVID-19 in Asia (4). In our patient with AMSAN, we observed fluctuations in symptom progression despite IVIG treatment. The patient’s condition stabilized and improved only after receiving PE treatment. At the follow-up, significant improvements in muscle strength were observed. The pathophysiological mechanism of GBS caused by COVID-19 is still unclear. For GBS triggered by SARS-COV-2, it has been hypothesized to be the result of cross-reactivity between the viral protein–associated gangliosides and peripheral nerve gangliosides leading to molecular mimicry (5). In contrast, others have suggested that GBS was not associated with COVID-19, as autoantibodies were not detected (6). In our patient with AMSAN, anti-ganglioside antibodies and GD1b IgG antibodies were not detected in the serum and CSF samples. The patient’s symptoms worsened once again after a brief period of improvement following immunoglobulin treatment, possibly indicating treatment-related fluctuations. It has been reported that one patient developed Guillain-Barré syndrome (GBS) after being infected with COVID-19, and treatment-related fluctuations (TRF) after receiving intravenous immunoglobulin (immunoglobulin) treatment. However, complete recovery was achieved after multiple rounds of IV-IG treatment. The mechanism behind this improvement following repeated treatment may be attributed to a rebound in the immune response (7).

Another hypothesis has suggested the direct invasion of peripheral nerves by SARS-COV-2. The olfactory nerve is the most common cranial nerve that is directly affected in this way (2). Recently, a different mechanism for the nerve damage presented in COVID-19-associated GBS has been proposed, suggesting primary facilitation through T-cell activation, release of inflammatory factors, and activation of macrophages, resulting in a hyperinflammatory response. Furthermore, a novel para infectious mechanism for GBS mediated by the generalized, hyperinflammatory response that occurs with COVID-19 has also been suggested, because the acute symptoms overlap with the onset of GBS, and autoantibodies were not detected (8). Our case supports this hypothesis. We have included this suggestion in the article. However, we did not carry out additional CD4/8 T lymphocyte tests. Further research is required to confirm and evaluate the common T-cell biomarkers using specialized blood panels. For our patient, serum and CSF ganglioside antibodies and GD1b IgG antibodies were negative. Moreover, weakness and paraesthesia in both the legs and arms were accompanied by a dry cough on admission. The peripheral nerve damage was severe and extensive including the spinal nerves, cranial nerves, and autonomic nerves. Importantly, the patient improved after immunomodulation therapy. It was emphasized that follow-up rehabilitation was also very important for returning to the community (9).

After admission, we performed a series of cerebral MRI examinations on this patient. When we compared and analyzed the images obtained during hospitalization and those obtained before disease onset, we were surprised that cerebral MRI showed an emerging SWI signal from microhaemorrhage in the right thalamus with a slightly enhanced T2 signal at the early stage of the disease. As the disease aggravated, the T2 hyperintensity in the right thalamus was significantly more intense and the coverage was larger. However, the T2 hyperintensity and T1 hypointensity of the right thalamus resolved when clinical symptoms improved. The abnormal T2 signal in the right thalamus evolved synchronously and dynamically with the changes in clinical symptoms. At the same time, in Figure 2O (SWI sequence from March 3, 2023), we observed changes in bilateral thalamic SWI, not only in the right thalamus, but also in support of the excessive immune response secondary to COVID-19. The presence of unilateral thalamus does not account for the patient’s initial presentation or the deterioration of symptoms. Thalamic lesions may not be directly linked to COVID-19 infection, but could potentially be a result of secondary immune reactions. To our knowledge, this is the first reported case of AMSAN with observable damage to intracranial neurons and blood vessels.

Bickerstaff brainstem encephalitis, a variant of GBS, is known to cause damage to the brainstem. However, it mainly affects the cranial nerves and the reticular activating system. Moreover, the condition does not have any indications on medical imaging, such as cerebral MRI (10). In our patient, the lesion was located in the right thalamus with microhaemorrhages and abnormal changes in neuronal signals. The etiological analysis suggested that an exaggerated immune response to the SARS-CoV-2 infection may have affected the cerebral small vessels and thalamic neurons, thereby resulting in microhaemorrhages as well as damage and changes in the neurons.

## Conclusion

AMSAN variants of GBS secondary to COVID-19 can cause severe peripheral nerve damage. The deterioration of symptoms in the patient after early immunotherapy may indicate treatment-related fluctuation (TRF) and could be attributed to immune rebound. Moreover, an excessive immune response post-COVID-19 infection may trigger concurrent damage to the central nervous system, indicating secondary harm to brain small blood vessels and nerve units. For suspected cases of GBS complicated by COVID-19, it is essential to conduct early brain MRI examinations in addition to routine peripheral nervous system evaluations to promptly detect any intracranial lesions. This facilitates appropriate immunotherapy and improves patient prognosis. Our study demonstrates that immunomodulatory therapy, such as the use of immunomodulatory drugs, can significantly alleviate both peripheral and central nervous system damage in patients with AMSAN variant GBS associated with COVID-19. This suggests that immunomodulatory drugs hold promising therapeutic value in addressing neurological symptoms related to COVID-19.

## Data availability statement

The original contributions presented in the study are included in the article/supplementary material, further inquiries can be directed to the corresponding author.

## Ethics statement

Ethical review and approval was not required for the study on human participants in accordance with the local legislation and institutional requirements. Written informed consent from the patients/ participants was not required to participate in this study in accordance with the national legislation and the institutional requirements. Written informed consent was obtained from the participant/patient(s) for the publication of this case report.

## Author contributions

All authors listed have made a substantial, direct, and intellectual contribution to the work and approved it for publication.

## Conflict of interest

The authors declare that the research was conducted in the absence of any commercial or financial relationships that could be construed as a potential conflict of interest.

## Publisher’s note

All claims expressed in this article are solely those of the authors and do not necessarily represent those of their affiliated organizations, or those of the publisher, the editors and the reviewers. Any product that may be evaluated in this article, or claim that may be made by its manufacturer, is not guaranteed or endorsed by the publisher.
